# A proof-of-concept study: advantages of the subxiphoid over the lateral intercostal approach

**DOI:** 10.1093/icvts/ivae067

**Published:** 2024-04-17

**Authors:** Gengxu He, Tong Yao, Lei Zhao, Hong Geng, Qiang Ji, Kun Zuo, Yuanzhi Luo, Kai Zhou

**Affiliations:** Department of Thoracic and Cardiovascular Surgery, The First Affiliated Hospital of Hebei North University, Zhangjiakou City, P.R. China; Department o the Cardiac Function, The First Affiliated Hospital of Hebei North University, Zhangjiakou City, P.R. China; Department of Thoracic and Cardiovascular Surgery, The First Affiliated Hospital of Hebei North University, Zhangjiakou City, P.R. China; Department of Thoracic and Cardiovascular Surgery, The First Affiliated Hospital of Hebei North University, Zhangjiakou City, P.R. China; Department of Thoracic and Cardiovascular Surgery, The First Affiliated Hospital of Hebei North University, Zhangjiakou City, P.R. China; Department of Thoracic and Cardiovascular Surgery, The First Affiliated Hospital of Hebei North University, Zhangjiakou City, P.R. China; Department of Thoracic and Cardiovascular Surgery, The First Affiliated Hospital of Hebei North University, Zhangjiakou City, P.R. China; Department of Thoracic and Cardiovascular Surgery, The First Affiliated Hospital of Hebei North University, Zhangjiakou City, P.R. China

**Keywords:** Video-assisted thoracic surgery, Anterior mediastinal tumor, Subxiphoid approach, Lateral intercostal approach

## Abstract

**OBJECTIVES:**

The study was designed to evaluate the superiority of the subxiphoid approach compared with the lateral intercostal approach during the operation and other perioperative indices.

**METHODS:**

Patients diagnosed with anterior mediastinal disease in our hospital between January 2018 and October 2019 were prospectively assigned to 2 groups; 1 group underwent the lateral intercostal approach and 1 group underwent the subxiphoid approach of video-assisted thoracoscopic surgery to resect the diseased tissue. The PaCO_2_, SaO_2_, PaO_2_ and circulation changes were recorded during the operation; the neutrophil-to-lymphocyte ratio and other perioperative outcomes, including clinical and surgical results, operating time, blood loss, postoperative complication and postoperative pain score were compared.

**RESULTS:**

A total of 59 patients diagnosed with an anterior mediastinal tumour or myasthenia gravis underwent a video-assisted thoracoscopic resection. Thirty-one patients were treated via the subxiphoid approach, and 28 patients were treated via the lateral intercostal approach. The PaCO_2_ increased significantly and the SaO_2_ remained stable in the subxiphoid group during the operation, whereas PaCO_2_ increased significantly and SaO_2_ decreased at the same time in the lateral intercostal group. Operations were more frequently interrupted for the hypoxia or circulation disturbance during the process of dissecting the thymus in the lateral intercostal approach. Compared with the lateral intercostal approach, patients treated via the subxiphoid approach experienced less inflammation and exhibited lower pain scores and shorter postoperative hospital stays. There were no significant differences in postoperative complications between the 2 groups. All of the patients recovered well when discharged.

**CONCLUSIONS:**

Our study results suggested that the subxiphoid approach has less of an influence on the pulmonary circulation than the lateral intercostal approach, that the whole procedure is safer and easier and that the subxiphoid approach may be the ideal choice for patients with anterior mediastinal disease.

## Graphical Abstract

**Figure:**
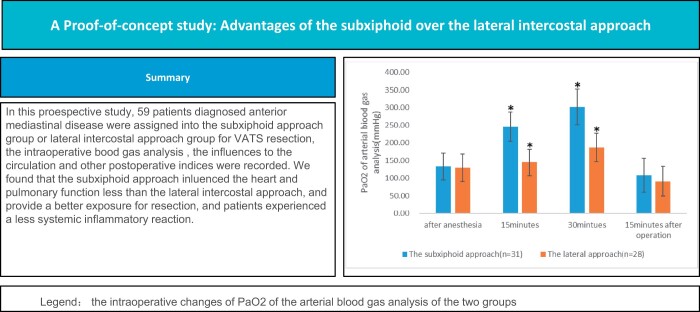

## INTRODUCTION

With the development of minimally invasive techniques, video-assisted thoracoscopic surgery (VATS) has been widely used in thymectomy for the treatment of myasthenia gravis and early-stage thymoma [1–4]. In patients with a wide distribution of ectopic thymic tissues in the anterior mediastinum, controversy still exists as to the optimal approach. The approaches for VATS thymectomy recently used in different centres included the lateral intercostal approach, the transcervical approach and the subxiphoid approach [2, 5–9]. Of these, the lateral intercostal approach is the most frequently performed. This approach does, however, have some disadvantages, including the exposure and the risk of intercostal nerve injury, which can result in postoperative chronic incision pain [10]. In recent years, the subxiphoid approach has raised more and more concerns. The advantages of the subxiphoid approach over the lateral intercostal approach include a sufficient operative view of the cervical and bilateral pleural cavities, less pain, a better cosmetic effect and an equivalent remission rate among seropositive myasthenia patients [6–10]. In this study, we prospectively evaluated the superiority of the subxiphoid approach over the lateral intercostal approach from another perspective.

## METHOD

### Ethical statement

This study was approved by the ethics committee of the First Affiliated Hospital of Hebei North University (2017021). Written informed consent about operative techniques and data-use agreements was obtained from all patients.

### Study design

Patients diagnosed with an anterior mediastinal tumour or myasthenia gravis with or without thymoma who underwent an operation were prospectively assigned to 2 groups—the subxiphoid group or the lateral intercostal group. Our plan was to enrolled 60 patients, 30 in each group. The 60 cards labelled ‘subxiphoid’ or ‘lateral’ were put into a box. Every patient was assigned before the operation by a random draw from the box. The initiation of anaesthesia and the operations were carried out by the same anaesthetist and the same surgeon. Patients who had had a previous thoracic surgical procedure, used opiates preoperatively, had a history of chronic pain syndrome and had radiologic evidence of invasion into the major vasculature were excluded.

### Surgical technique

#### Subxiphoid procedure

**Figure 1:**
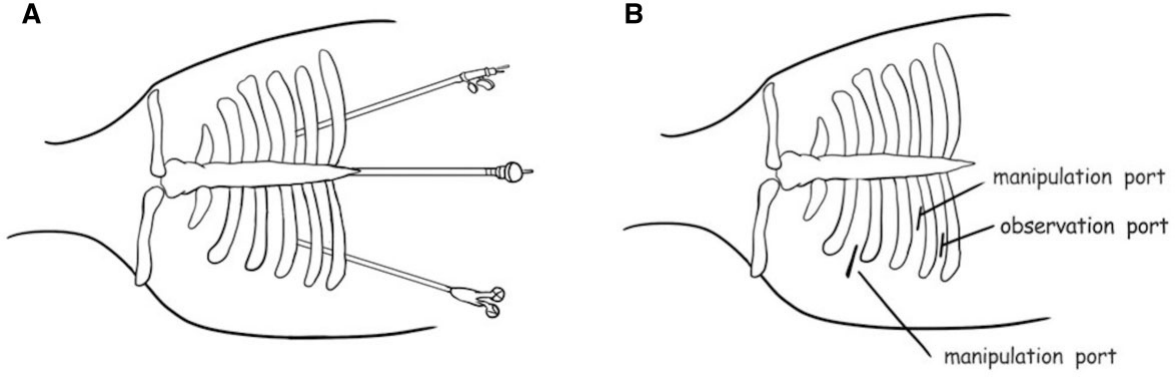
The incisions of the 2 approaches. (**A**) The subxiphoid and subcostal arch approach; (**B**) lateral intercostal approach (right).

**Figure 2:**
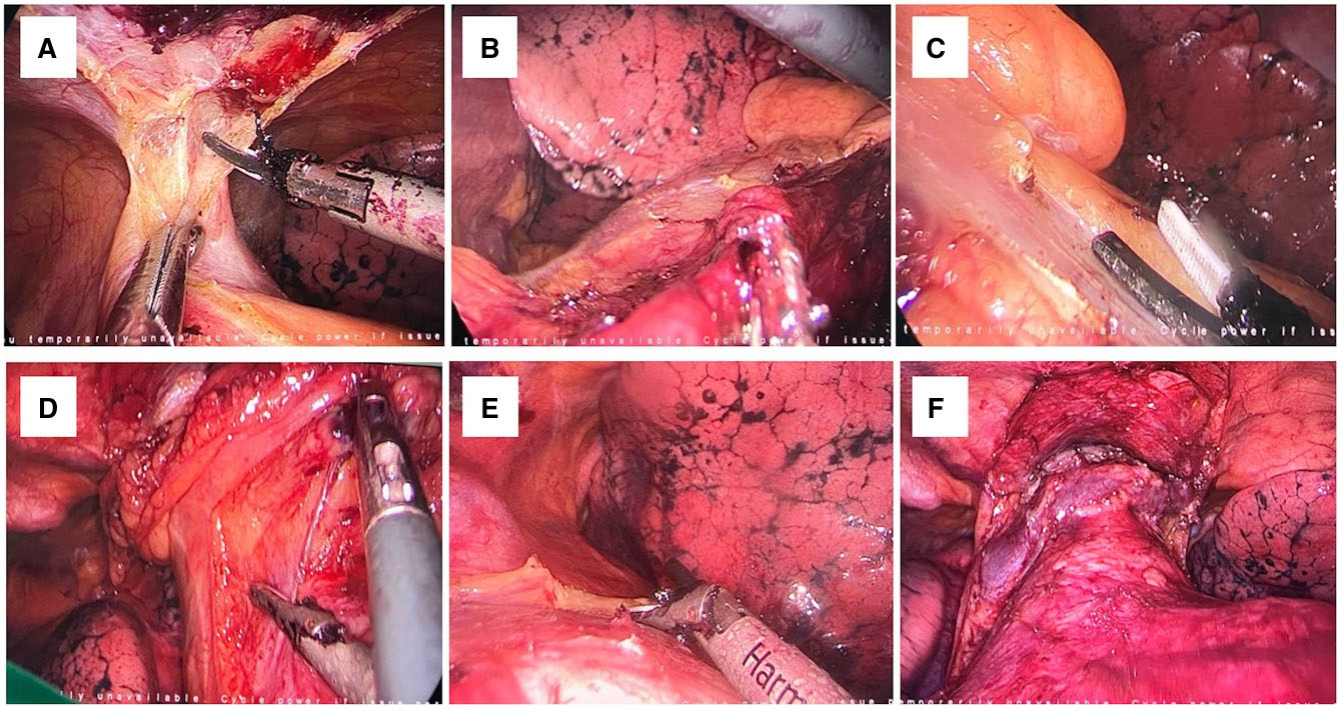
Procedure of the subxiphoid extended thymectomy. (**A**) Dissection of the anterior border of the thymus. (**B**) Right cardiac diaphragmatic angle. (**C**) Left cardiac diaphragmatic angle. (**D**) Exposure of the right phrenic nerve and dissection of its anterior adipose tissue. (**E**) Exposure of the left phrenic nerve and dissection of its anterior adipose tissue. (**F**) Postoperative view of the anterior mediastinum.

After being anaesthetized, all patients were intubated with a single-lumen endotracheal tube and placed in a supine position. The surgeon stood on the right side, and the assistant stood on the left side. A 2-cm transverse incision was made below the lower edge of the xiphoid as the observational port (Fig. 1A). The rectus abdominis muscle attached to the xiphoid was dissected, and the retrosternal space was bluntly separated with the surgeon’s finger as extensively as possible. Then two 5-mm thoracic ports were created under the bilateral costal arches at the midclavicular line, and a trocar was inserted into the working space, guided by the surgeon’s finger to avoid damage to the diaphragm and other organs. A 10-mm, 30° oblique-view rigid thoracoscope was introduced through the observation ports. Carbon dioxide with 8 to 10 mmHg of positive pressure was insufflated into the anterior mediastinum through the observation port. An ultrasonic scalpel was introduced through the left operating port, and thoracoscopic grasping forceps were introduced through the right operating ports. The bilateral pleural cavity was opened to create a bilateral pneumothorax. Then the anterior border of the thymus was gradually dissected along the retrosternal space vertically, to the upper margin of the thymus. In the right pleural cavity, the entire adipose anterior phrenic nerve was resected, which permitted full visualization of the innominate vein and the superior vena cava junction. The thymic vein draining into the innominate vein was dissected using the ultrasonic scalpel. In the left pleural cavity, all the surrounding adipose tissue along the anterior left phrenic nerve was removed to the junction of the left intrathoracic vein and left innominate vein (Fig. 2A–F). Finally, the upper part of the thymus in the superior space of the left innominate vein was dissected. The dissected whole thymus and any attached mediastinal fat designated for placement in the specimen bag were removed via the subxiphoid observation port. If the specimen was large, the transverse observation incision could be extended along the subcostal arch. A chest tube was inserted into 1 subcostal arch port for drainage postoperatively. The whole procedure was similar to that described by Qiang *et al*. [11] and Louqian *et al*. [12].

#### Lateral approach

The patients were anaesthetized after intravenous induction and intubated with a double-lumen endotracheal tube. The patients were then placed in a 30–45° semi-supine position (Fig. 1B). Depending on the position of the tumour, the anterior mediastinum was exposed via the right or left lateral intercostal incision. The observation port was inserted in the 6th intercostal space at the anterior axillary line, and this port was used for placing the trocar lens of the thoracoscope into the thoracic cavity and for placing the chest tube. One main manipulation incision was made in the 3rd intercostal space between the anterior axillary line and the midaxillary line. The ultrasonic scalpel and the thoracoscopic grasping forceps were introduced into the chest through this incision. The third incision was made in the 5th intercostal space at the midaxillary line if needed. In most cases, the thymus and adipose tissue in the mediastinum were resected, including the left and right cardiac diaphragm (Fig. 2B and C).

### Evaluation of the procedure

#### Intraoperative blood gas analysis

The arterial blood was collected at 4 time points: after initiation of anaesthesia; 15 min and 30 min after the beginning of the operation; and 15 min after the operation. The PaCO_2_, SaO_2_ and PaO_2_ values were recorded.

#### The haemodynamic index during the operation

During the operation, the haemodynamic status and the changes in blood pressure or cardiac arrythmia were recorded, and the interruptions to the operative procedure due to hypoxemia, haemodynamic disturbance and arrhythmia were recorded.

#### The inflammatory indices

Peripheral blood was obtained before the operation and on the first and the third postoperative days. The neutrophil-lymphocyte ratio was calculated by dividing the absolute count of neutrophils by that of the lymphocytes, and C-reactive protein (CRP) was detected routinely.

### Postoperative management and evaluation

The majority of patients were extubated immediately postoperatively. For pain management, all patients received patient-controlled analgesia with 0.2 mg/kg/h fentanyl and 0.1 mg/kg/h tramadol for 24–48 h, and postoperative pain scores were assessed at 6, 12, 24 and 48 h. Paracetamol oxycodone was administered as a rescue analgesic when requested by patients with a numerical rating score (NRS) greater than 4. The chest drain was removed when drainage was less than 100 ml/day with no air leakage within 48 h postoperatively. Patients were discharged after removal of the chest tube, when they were able to mobilize independently and when a chest computed tomography scan appeared normal.

## RESULTS

A total of 59 patients were enrolled during the study period; 31 patients had the subxiphoid procedure and 28 patients had the lateral intercostal approach, including 22 patients with a right lateral intercostal approach and 6 patients with a left lateral intercostal approach (Table 1). The 59th patient was assigned to the lateral intercostal approach, but the patient finally chose the subxiphoid approach because of the satisfactory results reported by other patients. The study was then terminated because no subsequent patients chose the lateral intercostal approach.

**Table 1:** The clinical data and operating information

Variables	Subxiphoid approach	Lateral approach	*P*-value
	(*n* = 31)	(*n* = 28)	
Clinical data			
Age (years)	49 ± 10.2	51 ± 11.2	0.95
Male	19	18	0.79
Tumour size (≥5 cm)	6	3	0.66
Operating time (min)	81 ± 23.4	75 ± 19.8	0.08
Blood loss (ml)	58(16.1)	60(14.3)	0.61
Conversion to thoracotomy	0	1	0.97
Delayed extubation	3(10.7)	2(8.9)	0.88
Total drainage (ml)	491 ± 173.4	509 ± 214.6	0.72
Hospital stay after operation, days	5 ± 3	7 ± 4	0.03
Pulmonary infection (n)	2	5	0.42
Phrenic nerve paresis (n)	3	2	0.88
Recurrent laryngeal nerve damage (n)	1	0	0.95
Postoperative pain			
24 h post-operation	4.2 ± 0.69	6.1 ± 0.79	<0.01
48 h post-operation	4.1 ± 0.53	6.3 ± 0.61	<0.01
72 h post-operation	4.4 ± 0.49	5.9 ± 0.89	<0.01
Pathological diagnosis			
Thymoma	19	21	0.62
Thymic hyperplasia	5	4	0.84
Thymic cyst	5	3	0.87
Thymic carcinoma	2	0	0.63

During the operation, the PaCO_2_ increased significantly after the bilateral artificial pneumothorax was created by insufflating carbon dioxide (CO_2_) with 8 mmHg positive pressure in the subxiphoid group, but the SaO_2_ did not decrease significantly. On the other hand, in the lateral intercostal group, the SaO_2_ decreased significantly and the PaCO_2_ increased at the same time the unilateral lung was ventilated (Table 2). The operative procedure for 9 patients was interrupted because of the hypoxemia, whereby the SaO_2_ decreased by more than 85% in the lateral intercostal group; it decreased in only 1 patient in the subxiphoid group. As for the haemodynamic effect, the operation was interrupted in 5 patients in the lateral intercostal approach group because of hypotension or cardiac arrhythmia due to cardiac decompression during the dissection of the cardiac diaphragm angle adipose from the opposite lateral intercostal incision. Comparatively, blood fluctuation at the beginning of the artificial pneumomediastinum occurred in only 1 patient in the subxiphoid approach group; the operative procedures for the other 30 patients went smoothly.

**Table 2:** Intraoperative gas analysis at different time points

Variables	Subxiphoid approach	Lateral approach	*P*-value
	(*n* = 31)	(*n* = 28)	
PaCO_2_			
After anaesthesia	36 ± 11	39 ± 10.2	0.28
15 min	46 ± 13.5	48 ± 12.9	0.56
30 min	42 ± 8.9	46 ± 13.2	0.17
15 min after the operation	38 ± 10.4	41 ± 10.8	0.28
PaO_2_			
After anaesthesia	133 ± 38	129 ± 39	0.69
15 min	246 ± 41*	145 ± 37	0.00
30 min	302 ± 51*	187 ± 40	0.00
15 min after the operation	109 ± 48	91 ± 42	0.13
SaO_2_			
After anaesthesia	98 ± 1.9	97 ± 2.8	0.1
15 min	95 ± 4.1	92 ± 7.9	0.06
30 min	96 ± 3.9	95 ± 4.1	0.06
15 min after the operation	95 ± 4.9	93 ± 4.6	0.11

**Figure 3:**
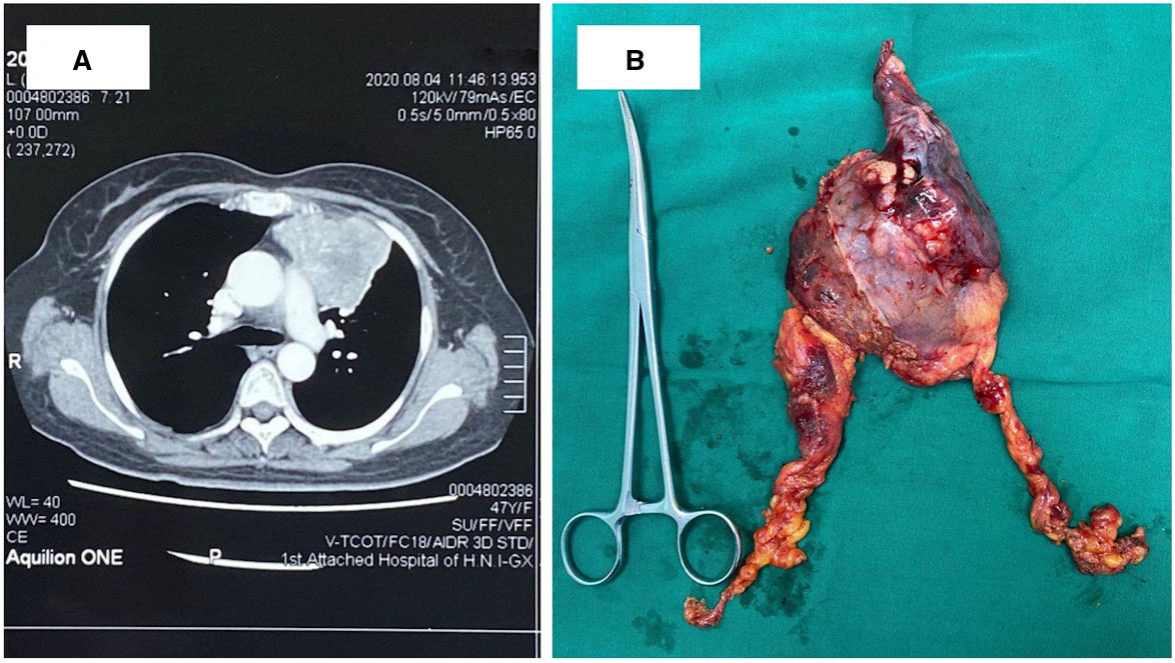
(**A**) Contrast enhanced computer tomograph shows a large tumour in the anterior mediastinum. (**B**) Specimen from the tumour and wedge-resected left upper lung tissue.

In the subxiphoid group, 6 patients had tumours larger than 5 cm, and 2 patients with stage III thymomas invading the right pulmonary … underwent concomitant right pulmonary wedge resection (Fig. 3). In the lateral intercostal approach, 3 patients had tumours larger than 5 cm, and 1 patient was converted to an upper sternotomy because the tumour adhered to the innominate vein. We found that exposure of the whole body of the anterior mass is more efficient and easily performed in the subxiphoid group.

The postoperative inflammatory indices (NLR and high sensitivity-CRP) were significantly higher in the lateral intercostal group compared with the subxiphoid group during the postoperative period (Table 3).

**Table 3:** Postoperative inflammatory indices of the 2 groups

Variables	Subxiphoid approach	Lateral approach	*P*-value
	(*n* = 31)	(*n* = 28)	
hs-CRP			
Pre-operation	1.21 ± 0.23	1.31 ± 0.27	0.12
D1 post-operation	3.2 ± 1.23	4.9 ± 1.86	0.00
D5 post-operation	2.2 ± 1.23	3.1 ± 1.32	0.00
D7 post-operation	1.34 ± 0.48	2.1 ± 0.69	
NLR			0.81
Pre-operation	2.32 ± 1.58	2.41 ± 1.39	0.00
D1 post-operation	5.41 ± 2.08	12.89 ± 3.78	0.00
D5 post-operation	3.44 ± 1.98	7.32 ± 2.15	0.00
D7 post-operation	2.44 ± 1.43	4.36 ± 1.62	0.00

D: day; hs-CRP: high-sensitivity C-reactive protein; NLR: neutrophil-lymphocyte ratio.

**Figure 4:**
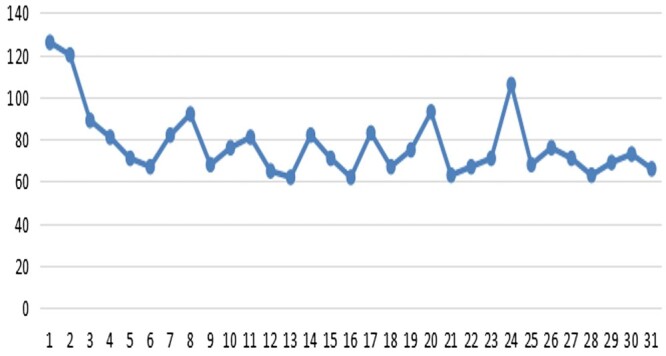
The learning curve of the subxiphoid approach (the operating time of the subxiphoid approach from the first patient to the 31st patient).

There were no significant differences in the mean operative times or in the median intraoperative blood loss. In fact, if we excluded the 6 patients with tumours larger than 6 cm whose operations took more time than the other patients in the subxiphoid group, the mean operative time was less than that of the lateral intercostal group. At the same time, we recorded the learning curve of an experienced thoracic surgeon (who could carry out routine thoracoscopic operations independently) in learning the subxiphoid approach technique. The surgeon became familiar with the technique after a short practice period (Fig. 4), and then the mean operative time was significantly shorter than that of the lateral intercostal approach.

All 59 patients recovered without incident. In the subxiphoid group, the phrenic nerves encapsulated in the tumour were damaged during the dissection of the tumour in 3 patients, and 1 patient experienced left recurrent laryngeal nerve damage because the dissection of the tumour adhered to the aorta-pulmonary artery window, but recovered after 3 months. In the lateral intercostal group, the phrenic nerves of 2 patients were damaged during dissection of the tumour.

There were statistically significant differences between the 2 groups with respect to postoperative pain [NRS for 24 h, 2.9 (2–4) vs 4.1 (3–5), *P* = 0.02; NRS for 48 h, 2.6 (2–3) vs 4.3 (3–5), *P* = 0.01]. In addition to the primary end point, patients treated with the subxiphoid approach had less pain compared with those undergoing the lateral approach.

## DISCUSSION

Considering the wide distribution of ectopic thymic tissues in the anterior mediastinum, most thoracic surgeons recommend resection of all the soft tissues in the prevascular plane of the anterior mediastinum between the 2 phrenic nerves, especially for patients with gravis myasthenia, regardless of the thoracoscopic or open approaches [10, 13, 14]. In open surgery, the transsternal approach is recognized as the standard procedure for an extended thymectomy. However, different approaches have emerged for thoracoscopic thymectomy, which include a unilateral (right or left side), bilateral and transcervical or a combination approach [2, 11, 15–23]. The subxiphoid approach has recently been used with greater frequency, and its superiority has been more and more accepted.

We analysed the arterial blood gases at different times to determine the intraoperative gas exchange and the haemodynamic changes occurring during the whole procedure to compare the influence of the 2 approaches. We found that PaCO_2_ levels increased significantly and remained in a stable and accepted range during the whole procedure in the subxiphoid group, but the SaO_2_ level did not change significantly. On the other hand, an increase in PaCO_2_ occurred along with a decrease in SaO2 during unilateral lung ventilation in the lateral intercostal group, and the operations of 7 patients were interrupted to allow the anaesthetist to ventilate the bilateral lung to alleviate the hypoxia. These changes indicated that, although the subxiphoid approach with an artificial pneumothorax on both pleural cavities did not affect the pulmonary ventilation and ventilation/blood flow ratio, the lateral intercostal approach, unilateral lung ventilation and the lateral position affected the pulmonary ventilation and ventilation/blood flow ratio more severely, and some patients needed to have the double lumen tracheal tube repositioned or to have bilateral lung ventilation for a short time during the procedure. As for the influences on circulation, 5 patients experienced a decrease in blood pressure and arrythmia when the surgeon dissected the adipose fat tissue of the contralateral angle of the cardiac diaphragm in the lateral intercostal group, but only 1 patient had a decrease in blood pressure when a pneumomediastinum was created at the beginning of the operation in the subxiphoid group. Exposure of the whole body of the anterior mediastinum tumour and the thymus was easier and clearer through the subxiphoid approach, so there were fewer influences on the circulation during the whole procedure. But in the lateral intercostal approach, as has been reported in some articles, it is difficult to expose the contralateral side and to remove all the mediastinal fat tissue, especially the angle of the cardiac diaphragm. During the exposure of the contralateral side, the tissues of the heart are influenced severely and may even cause hypotension or arrythmia.

The postoperative inflammatory indices (neutrophil-lymphocyte ratio and CRP) were significantly higher in the lateral intercostal group during the postoperative period, which means that intraoperative unilateral lung ventilation in the lateral intercostal group induces a more severe inflammatory reaction than in the subxiphoid group. Zhang also reported that the white blood cell counts and CRP levels were lower in the subxiphoid group than in the lateral intercostal group on postoperative day 1 [12].

The reported thoracoscopic approaches for thymectomy are associated with several limitations. In the unilateral approach, it is difficult to expose the contralateral side and to remove all the mediastinal fat tissue [24]. Although the bilateral approach provided adequate exposure of the anterior mediastinum, more incisions were needed, which may increase operative trauma and postoperative pain [15–17]. Hsu [6, 25], Zielinski [18, 24, 26] and Suda [22, 23] reported using a thoracoscopic thymectomy partially or entirely via subxiphoid incisions but that a port incision using a Kent retractor to lift the sternum [25, 27] and the interference of the instruments in the single subxiphoid incision [12, 22, 23] are major limitations. Compared with those approaches, the exposure of all the anterior mediastinum tissue, which represents the advantages of the subxiphoid and subcostal arch approaches, facilitates the removal of fat tissue and minimizes the chance of surgical injury such as accidental vessel laceration or contralateral phrenic nerve injury [27]. In our study, more patients with large tumours could be resected through the subxiphoid and subcostal arch approach. At the same time, the combined lesion could be handled for the excellent exposure. Second, the free use of the double lumen endotracheal tube avoided the need to regulate the position of the tube during the operation. Patients with severe lung disease or poor lung function intolerant to unilateral lung ventilation and young patients unable to accommodate the smallest double-lumen tube could also be treated [11]. Third, the procedure resulted in less postoperative pain by avoiding injury to the intercostal nerve. Therefore, the postoperative pain score was far less compared with that from the lateral intercostal approach [27].

An experienced thoracic surgeon will require less time to master this technique. We collected the operating times from the first operation and found only a short learning curve was needed to master this technique. We did not find a significant difference in the operating times. There were more patients with tumours larger than 5 cm, and it took more time to dissect the larger tumours, which adhered to surrounding tissue. When we excluded these patients, we found that the operating time was significantly less in the subxiphoid group, as reported by other authors [9, 28].

These results indicate that the subxiphoid approach has less influence on the pulmonary circulation than the lateral intercostal approach and that the whole procedure is safer and easier and that the subxiphoid approach may be the ideal choice for treating the anterior disease.

### Limitations

Our study has some limitations. First, it is a small cohort and a single-institution prospective study, so the study design, that is, the heterogeneous population and the surgeon’s bias, may influence the results. Second, the follow-up time is short. We only monitored the intraoperative cardiopulmonary function and the perioperative results. We did not investigate the long-term results. Third, we initially planned to recruit 60 patients, but in the end we enrolled only 59 patients. No additional patients chose to accept the lateral intercostal approach because of the satisfactory results obtained with the subxiphoid approach.

## Data Availability

The data are available from the corresponding author upon receipt of a reasonable request.

## ABBREVIATION

NRS: Numerical rating score
VATS: Video-assisted thoracoscopic surgery
